# Complete genome of a dengue virus serotype 4 strain from Amazonas, Brazil

**DOI:** 10.1590/0074-02760150416

**Published:** 2016-02

**Authors:** Valdinete Alves do Nascimento, Victor Costa de Souza, Felipe Gomes Naveca

**Affiliations:** 1 Fundação Oswaldo Cruz, Instituto Leônidas e Maria Deane, Manaus, AM, Brasil; 2 Universidade Federal do Amazonas, Programa de Pós-Graduação em Saúde, Sociedade e Endemias na Amazônia, Manaus, AM, Brasil

**Keywords:** Brazilian Amazon, dengue virus, DENV-4, viral genome, next-generation sequencing

## Abstract

Dengue virus (DENV) infections represent a significant concern for public health worldwide, being considered as the most prevalent arthropod-borne virus regarding the number of reported cases. In this study, we report the complete genome sequencing of a DENV serotype 4 isolate, genotype II, obtained in the city of Manaus, directly from the serum sample, applying Ion Torrent sequencing technology. The use of a massive sequencing technology allowed the detection of two variable sites, one in the coding region for the viral envelope protein and the other in the nonstructural 1 coding region within viral populations.

Dengue fever is considered one of the most significant public health issues worldwide, being endemic in more than 100 countries (WHO 2014). The etiologic agent of dengue fever, dengue virus (DENV), belongs to the genus Flavivirus, family Flaviviridae. Four distinct serotypes denoted DENV-1 to DENV-4 are recognised (Gubler 1998), and each serotype may be further separated into different genotypes. To DENV-4, four genotypes have been described (Klungthong et al. 2004, Weaver & Vasilakis 2009).

In Brazil, the first reported cases of the disease occurred in the city of Boa Vista, state of Roraima, during an outbreak in 1982 by serotypes DENV-1 and DENV-4 (Travassos da Rosa et al. 1982). Nevertheless, the first recognised dengue epidemics in Manaus, the capital of state of Amazonas (AM), were reported during 1998-1999 (de Figueiredo et al. 2004). Unlike the other serotypes, DENV-4 was not detected again in Brazil until 2008, when it was reported in autochthonous cases occurring in AM (de Figueiredo et al. 2008).

In this paper, we report the complete genome sequencing of a DENV-4 sample, named as BR005AM_2011, one of the first samples of the genotype II identified in AM. The present data was obtained by next-generation sequencing on Ion Torrent Personal Genome Machine (PGM™).

Firstly, a serum sample was submitted to total RNA extraction and complementary DNA synthesis followed by a semi-nested polymerase chain reaction (PCR) protocol (Lanciotti et al. 1992) to confirm the DENV-4 serotype. After that, the whole genome was amplified using eight sets of primers to generate overlapping amplicons spanning the entire viral genome (Naveca et al. 2012).

The library for Ion Torrent - PGM™ System was prepared from an equimolar solution of eight sets of amplicons generated by the PCR reaction. Further, those amplicons were enzymatically cleaved and barcoded adapters were linked to DNA fragments, generating the libraries. Moreover, libraries were amplified by emulsion PCR with ION PGM Template OT2 200 kit.

Sequencing was performed on a 314v2 chip generating 954,446 reads. After quality analysis, 484,647 reads were used for assembly, with an average size of 221 bases. The reads were analysed on Geneious Pro 7.1.0 (Biomatters, Ltd), employing Geneious’s map to reference assembler tool, with 10 interactions and DENV-4 reference sequence, available in GenBank under accession NC_002640.1.

The whole genome of the BR005AM_2011 sample, containing 10,649 nucleotides with 47.2% of GC content, was obtained with a mean coverage of 1,181x. In addition to consensus sequence, the next-generation sequencing allowed to analyse the existence of variable sites in subpopulations within sequences that differ from the consensus sequence, which may aid in studies of viral evolution. We also determinate the full-genome sequence of BR005AM_2011 by capillary sequencing and no differences were found regarding to the consensus sequence.

Analysis of variable sites was held at Geneious Pro 7.1.0 with the Variations/SNPs tool using the criteria of minimum coverage 250x and minimum variant frequency of 0.1%. The analysis showed the existence of two variable sites, one at the coding region of the viral envelope protein and the other in the nonstructural 1 coding region (Table).

**TABLE:** Variable sites identified by next-generation sequencing and analysis of variants

Site^*a*^	QV^*b*^	Cov^*c*^	Maj^*d*^	Var^*e*^	Freq (%)	p^*f*^	SB (50%)	Type	Pos^*g*^	aa^*h*^
1887	31	295	C	T	18.3	1.9 x 10^-108^	0.34	NS	**t** at	His593Tyr
2632	30	456	A	G	19.3	2.6 x 10^-140^	0.54	NS	c **g** a	Gln844Arg

*a* : according to the GenBank RefSeq NC_002640.1;*b* : quality value (QV) score for base call;*c* : sequencing coverage (Cov) at position;*d* : nucleotide found in the majority (Maj) sequence;*e* : nucleotide found in the variant (Var) sequence;*f* : variant p-value; *g* : position at the codon (bold); *h* : position according to the GenBank RefSeq NP_073286.1; aa; amino acid; Freq: frequency; NS: nonsynonymous; SB: strand-bias.

The viral genotype was characterised by phylogenetic analysis of the complete genome, using the neighbor-joining method and the Tamura-Nei genetic distance model. A data set of DENV-4 complete genomes encompassing representatives for each genotype was used as a reference (Figure). The BR005AM_2011 sequence was grouped with others DENV-4, genotype II, which were detected in Venezuela and Colombia.

**Figure:**
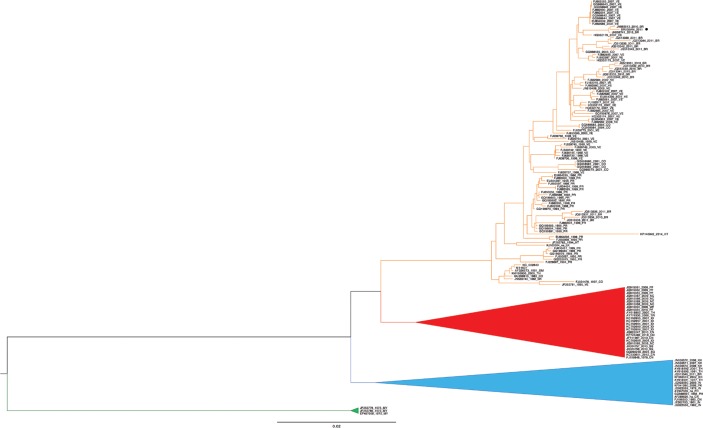
Phylogenetic reconstruction of dengue virus (DENV) isolate BR005AM_2011. A neighbor-joining tree was constructed with 148 taxa (complete CDS) representing the four DENV-4 genotypes and 1,000 bootstrap replicates. Genotypes I (GI), III (GIII), and sylvatic (outgroup) are represented by blue, red, and green cartoons, respectively. Genotype II is represented by an orange clade and sample BR005AM_2011 from the city of Manaus, state of Amazonas, Brazil, is highlighted by a dark circle.

In summary, the approach applied in the present study using Ion Torrent semiconductor sequencing may be easily used to obtain the complete genome of dengue or any other virus with similar genome size. Furthermore, several samples can be sequenced together and in-depth coverage, allowing a variety of analyses that may help understand the mechanisms related to viral evolution.

The whole genome of DENV-4 isolate BR005AM_2011 is available in GenBank under accession KT794007.
